# CMR assessment of hypertrophic cardiomyopathy with restrictive phenotype

**DOI:** 10.1186/1532-429X-17-S1-P299

**Published:** 2015-02-03

**Authors:** Minjie Lu, Shihua Zhao, Yan Zhang, Bailin Wu, Jing An

**Affiliations:** 1 Magnetic Resonance Imaging, Fuwai Hospital, Beijing, China; 2 Radiology, the Second Hospital of Hebei Medical University, Shijiazhuang, China; 3 MR Collaborations NE Asia, Beijing, China

## Background

Hypertrophic cardiomyopathy is a heterogeneous myocardial disorder with a broad spectrum of clinical presentation and morphologic features. Recent reports indicated that some patients with restrictive cardiomyopathy. Comprehensive cardiac magnetic resonance imaging of the restrictive phenotype in HCM patients has not been fully evaluated. The purpose of this study was to investigate the CMR characteristics of hypertrophic cardiomyopathy (HCM) with restrictive phenotype.

## Methods

19 patients of HCM with obviously restrictive characteristics and 19 patients with non obstructive HCM, matched with age and gender, were collected. The differences in clinical features, CMR morphological characteristics, and the function parameters were retrospectively compared of the two groups. The paired sample t test and X^2^ test/Fisher's exact probability method were used for statistical analysis.

## Results

Restrictive phenotype of patients with HCM have more severe clinical conditions including sustained atrial fibrillation, pericardial effusion, and lower heart function classifications compared with controls; The left and right atrium diameter were 55.79±5.34 mm and 61.33±11.05 mm, which were significantly greater than the controls (p<0.001); The segments with late gadalinum enhancement were 7.68±2.98, which were significantly more than that in controls (5.10 ±2.77,p = 0.008). The left ventricular end-diastolic volume index, the cardiac index, and the left heart ejection fraction of patients with restrictive phenotype were all significantly less than those of the controls.

## Conclusions

Restrictive phenotype is a special subtype of HCM. The CMR features include mild-to-moderate left ventricular hypertrophy, huge atrium, normal or small left ventricular, pericardial effusion and a wide range of late gadalinum enhancement, often with severe clinical symptoms and poor prognosis. MRI has important application value in the diagnosis of the disease.

